# Prevalences of SARS-CoV-2 RNA and anti-SARS-CoV-2 among at-risk populations in Chiang Mai and Lamphun provinces, Thailand, during November 2020–January 2021

**DOI:** 10.1371/journal.pone.0263127

**Published:** 2022-02-02

**Authors:** Siriluk Takalay, Nicole Ngo-Giang-Huong, Wanlee Kongnim, Paporn Mongkolwat, Porntip Phoseng, Nantawan Wangsaeng, Sayamon Hongjaisee, Bordin Butr-Indr, Khajornsak Tragoolpua, Gonzague Jourdain, Sakorn Pornprasert, Woottichai Khamduang

**Affiliations:** 1 Department of Medical Technology, Faculty of Associated Medical Sciences, Chiang Mai University, Chiangmai, Thailand; 2 MIVEGEC, Univ. Montpellier, CNRS, IRD, Montpellier, France; 3 Associated Medical Sciences (AMS)-PHPT Research Collaboration, Chiangmai, Thailand; 4 Research Institute for Health Sciences, Chiang Mai University, Chiangmai, Thailand; 5 Infectious Diseases Research Unit (IDRU), Faculty of Associated Medical Sciences, Chiang Mai University, Chiangmai, Thailand; Konkuk University, REPUBLIC OF KOREA

## Abstract

Non-healthcare workers with a high potential for exposure to severe acute respiratory syndrome coronavirus type 2 (SARS-CoV-2) may contribute to the virus spreading. Data among asymptomatic and high exposure risk populations is still scarce, in particular Chiang Mai and Lamphun provinces, Thailand. We conducted a cross-sectional observational study aiming to assess the prevalence of SARS-CoV-2 RNA positivity, anti-SARS-CoV-2 IgM/IgG, and potential associated factors among asymptomatic/mild symptomatic individuals with a high exposure risk in Chiang Mai and Lamphun provinces, during the second wave of outbreak in Thailand (November 2020–January 2021). Socio-demographic data was collected through an on-line questionnaire prior to collection of nasopharyngeal/throat swab samples and blood samples tested for SARS-CoV-2 RNA (DaAn Gene, China) and anti-SARS-CoV-2 IgM/IgG antibodies (commercial lateral flow immunoassays), respectively. Univariable and multivariable logistic regression analysis were used to analyze associated factors. None of 1,651 participants were found positive for SARS-CoV-2 RNA (0%, 95% confidence intervals, CI: 0–0.2). Fourteen were positive for anti-SARS-CoV-2 IgM/IgG antibodies (0.9%, 95% CI: 0.5–1.4), including 7 positives for IgM and 7 positives for IgG (0.4%, 95% CI: 0.2–0.9). Being over 50 years old was independently associated with virus exposure (OR: 5.8, 95% CI: 1.0–32.1%, p = 0.045). Despite high exposure risk, no current infection was found, and a very high proportion was still susceptible to SARS-CoV-2 infection and would clearly benefit from vaccination. Continuing active surveillance, rolling out of vaccination and monitoring response to vaccine will help better control the COVID-19 spread.

## Introduction

The emergence of a new human coronavirus, severe acute respiratory syndrome coronavirus 2 (SARS-CoV-2), in late 2019 has sparked an explosive global pandemic of Coronavirus Disease 2019 (COVID-19) [1, 2]. Incubation period after virus acquisition was about 6.4 days [3]. Manifestations of COVID-19 vary from asymptomatic to fatal. The proportions of asymptomatic individuals ranged between 20–75% among COVID-19 cases according to different study groups, countries and the mean age of studied population [4, 5]. Common clinical manifestations include fever, dry cough, dyspnea, myalgia and fatigue. Some cases may develop an acute respiratory distress syndrome (ARDS), shock, and multiple organ failure leading to death [6, 7]. The mortality rate of COVID-19 in the most affected countries was about 0.5–9% [8]. The majority of deaths occurred mostly in elderly people aged over 60 years old and people with underlying diseases such as cardiovascular disease, diabetes mellitus, hypertension and malignancy [9].

The disease rapidly spread in China and soon after in other countries, raising a major global concern. It was then declared as a pandemic on March 11, 2020 [10] and has remained a problem since the first outbreak due to an uncontrolled spread in various countries and limited access to effective vaccines. From the beginning of the outbreak to prior the present study commenced (August 31, 2020), nearly 25 million cases worldwide were confirmed for SARS-CoV-2 infection, and over 0.8 million deaths were reported by World Health Organization (WHO) [11]. Thailand was among the first countries where report imported cases from China in January 2020 [10, 12]. The initial outbreak occurred in March 2020, originating from boxing stadiums and drinking venues in the capital city [13], then spread to the whole country. Until August 31, 2020, over 3,400 SARS-CoV-2 infected cases were reported with 58 deaths throughout the country [14]. Chiang Mai and Lamphun provinces are located in the Northern region of Thailand and they are characterized by a strong tourist industry and intense industrial activities, respectively. Due to these activities, many visitors travel to these two provinces with the risk of spreading further COVID-19 outbreak. During the time of conducting the study, the second wave of COVID-19 outbreak has occurred. Some infected cases were identified among smugglers from Myanmar in Chiang Mai province which corresponded with the small rising cases of COVID-19 in Myanmar. Therefore, the surveillance must be strengthened in individuals who has a risk history.

Individuals with high-risk exposure to SARS-CoV-2 include people who traveled from an outbreak area or worked in close contact with people or a crowd such as healthcare workers, delivery men, customer service staff, garbage collectors, municipal waste collectors, *etc*. Even asymptomatic or mild symptomatic, these individuals might serve as a reservoir and transmit virus to susceptible people and may play a significant key role in viral spreading. Indeed, asymptomatic people, *i*.*e*. healthcare workers and travelers from an outbreak area, have been shown to transmit SARS-CoV-2 to others [15–17]. Thus, the strategies to control infection in these groups had been considered. A priority identification of new COVID-19 cases is one of strategies to control the virus spread/outbreak and remains a challenge. A proactive COVID-19 test strategy can be effectively implemented in a real-life situation [18]. To identify individuals with SARS-CoV-2 infection, real-time reverse transcriptase polymerase chain reaction (rRT-PCR) is commonly used as a standard method for detecting SARS-CoV-2 RNA in specimens. SARS-CoV-2 IgG/IgM antibody testing can be used as a part of screening tests to identify individuals who have been exposed to the virus [19].

Data on SARS-CoV-2 prevalence and seroprevalence in Thailand originated from infected individuals (hospitalized and recovered COVID-19 patients), close contacts with recovered COVID-19 patients and healthcare workers is available [20–23]. Data among asymptomatic/mild symptomatic and high exposure risk populations is still scarce, in particular Chiang Mai and Lamphun provinces, Thailand. We used an outreach and contactless care service system to assess the prevalence of SARS-CoV-2 RNA positivity in nasopharyngeal/throat specimens and anti-SARS-CoV-2 IgM/IgG antibodies seroprevalence and the associated factors among at-risk populations in Chiang Mai and Lamphun provinces, Thailand, around the second wave of COVID-19 outbreak.

## Materials and methods

### Study population and data collection

People living in Chiang Mai and Lamphun provinces, Thailand, were recruited between November 2020 and January 2021. The populations were targeted at individuals with risk exposure to SARS-CoV-2 infection presenting no or mild symptoms and had a difficulty to access the COVID-19 testing. Mild symptoms were defined as the presence of at least one of the following: fever least or chills, cough, shortness of breath or difficulty breathing, fatigue, muscle or body aches, headache, new loss of taste or smell, sore throat, congestion or runny nose, nausea or vomiting, diarrhea, and no need of hospitalization [24]. The at-risk populations in this study included individuals who were at-risk to expose to SARS-CoV-2 infection, which may relate to their behaviors, household being, or occupations. These population include migrant workers, delivery men, customer service staff, public-facing workers, municipal waste collectors and others. Healthcare workers who were already supported for COVID-19 testing by the government were excluded. This study was a part of a healthcare service that we have provided to the community with free-of-charge to control the COVID-19 outbreak. In addition, participants were however informed (either documental or orally) and provided their consent via a written form or an online application before registration for sample collection. Data were collected using an on-line or paper questionnaires with five sections covering socio-demographic characteristics, health information, history of travel, medical history, and symptoms related COVID-19. This study was approved by the Ethic Committee of the Faculty of Associated Medical Sciences, Chiang Mai University (AMSEC-63EX-017). The need for the minor’s consent was waived by the Ethics Committee.

### Clinical specimens

Nasopharyngeal/throat swab samples and blood samples were collected from participants through an outreach and contactless care service system. All specimens were strictly collected by well-trained health care personnel according to biosafety standard precautions. Nasopharyngeal/throat swabs were transferred into viral transport media for SARS-CoV-2 RNA testing. Ethylenediaminetetraacetic acid (EDTA) blood samples were collected for SARS-CoV-2 IgM/IgG antibodies testing. All samples were transported under 2–4°C condition to the laboratory of the faculty of Associated Medical Sciences, Chiang Mai University—Institut de Recherche pour le Développement (AMS CMU-IRD) collaboration for further processes within a few hours. All clinical specimen samples were processed in biosafety level-2 enhanced (BSL-2 enhanced) facilities with full personal protective equipment.

### Detection of SARS-CoV-2 RNA using real-time RT-PCR

The nasopharyngeal/throat swab samples were processed for RNA extraction using QIAamp Viral RNA Mini Kit (QIAGEN, Germany) or Nucleic Acid Extraction Kit (Zybio, China), according to the manufacturer’s recommendations. SARS-CoV-2 RNA was detected by real time RT-PCR assay using a commercial test kit targeting at *ORF1ab* and *N* Genes and together with human endogenous gene served as internal control (DaAn GENE Co., Ltd.) which were operated on the automated *ab*CyclerQ instrument (ATI Biotech, Singapore). This study, the sample was considered as positive if the cycle threshold (Ct) values ≤40, according to the manufacture’s recommendation. The positive sample was further confirmed with in-house COVID-19 test kits targeting *RNA-dependent RNA polymerase (RdRp)* and *N* genes using the protocol available from the Department of Medical Sciences of Thailand and the WHO.

### Detection of anti-SARS-CoV-2 antibodies using immunochromatography assay

Blood samples of participants were collected in the EDTA tube and then were centrifuged to obtain plasma. Initial serological testing was performed using rapid tests, COVID-19 IgG/IgM Device (Prestige, UK; 100% sensitivity for IgG and 85% for IgM; 98.0% specificity for IgG and 96.0% for IgM) or 2019-nCoV Ab Test (INNOVITA, China; 87.3% sensitivity and 100% and specificity). The positive samples were confirmed by SARS-CoV-2 Rapid Antibody Test (SD BIOSENSOR, Korea; 92.59% sensitivity and 98.65% specificity) and 2019-nCoV IgG/IgM Rapid Test Cassette (ACRO, U.S.A, 96.9% sensitivity and 98.2% specificity). Sensitivity and specificity are described in the product package insert. Antibodies results were considered according to the customized algorithm. Briefly, negative on the screening test (either Prestige or INNOVITA) was reported as negative. Positive on the screening test was confirmed with other test kits (SD BIOSENSOR and ACRO). Samples were considered as negative if confirmed test kits revealed negative results.

### Statistical analysis

The sample size of the study population was calculated based on previous reports data during the first outbreak of COVID-19 in Thailand. We estimated that 1% of populations were infected with SARS-CoV-2, with 0.5% acceptable error, 95% confidence level. The sample at least 1,521 samples were recruited into the study. Values for categorical data were presented as percentages and values for continuous variables were presented as median with interquartile range (IQR). Continuous variables were dichotomized using common cut-off values. The proportions of individuals positive for SARS-CoV-2 RNA or SARS-CoV-2 IgM/IgG antibodies were presented as percentage, along with 95% confidence intervals (CI). Univariable analysis was performed using logistic regression to identify factors potentially associated with an exposure to SARS-CoV-2, *i*.*e*. anti-SARS-CoV-2 IgM/IgG antibodies positive. Variables with a *p*-value lower than 0.250 in the univariable analysis were further entered into a multivariable analysis, and a backward elimination procedure was used to identify factors independently associated with SARS-CoV-2 IgM/IgG antibodies positivity. Data was analyzed using STATA 14.1 software (StataCorp, College Station, TX, USA). Statistically significant was considered if *p*-values less than or equal to 0.05.

## Results

### Characteristics of study population

A total of 1,651 participants from at-risk populations were recruited. Their characteristics are presented in Tables 1 and 2. The median age was 35 years old (IQR: 28–44), ranging from 17–85 years old. The ratio between females and males was 1.1. Eight women were pregnant. About 72% had resided in Chiang Mai province and 26% in Lamphun province. Almost two-third of participants were Thai and one-third were Burmese. In terms of education, participants who “Never attended school” represented the highest proportion (31.6%). There were a variety of occupations, *i*.*e*. general laborer/freelance, customer service representative and employees of companies/private organizations which represented at 30.0%, 24.6% and 14.2%, respectively. About 94% had worked in a place with colleagues and 58% worked in contact with customers. More than a half (62.2%) had Body Mass Index (BMI) in the normal range (18.5–24.9 kg/m^2^) with a median of BMI at 22.9 kg/m^2^ and one-third had BMI above 25 kg/m^2^. During the study period, only 3% of participants had traveled within the last two weeks before registration. Furthermore, 197 (6.8%) participants had a history of medical conditions including high blood pressure/cardiovascular disease and diabetes. 21.8% (358/1,646) reported mild symptoms related to COVID-19, including runny nose (10.4%), sore throat (6.5%) and fatigue (5.7%) (Table 2).

**Table 1:** Baseline socio-demographic characteristics of study population.

Baseline characteristics	Total		Female		Male	
	N	median (IQR) or n (%)	N	median (IQR) or n (%)	N	median (IQR) or n (%)
**1. Socio-demographic characteristics**						
Age (years old)	1,651	35 (28–44)	852	35 (28–44)	791	35 (27–43)
Sex	1,651					
Female		852 (51.7)				
Male		791 (48.0)				
Non-binary		5 (0.3)				
Missing		3				
Pregnant	820	8 (1)	820	8 (1)		
Residence	1,601		826		767	
Chiang Mai		1,161 (72.5)		591 (71.6)		564 (73.5)
Lamphun		417 (26.1)		222 (26.9)		193 (25.2)
Others		23 (1.4)		13 (1.6)		10 (1.3)
Country of birth	1,647		851		788	
Thailand		1,070 (65.0)		564 (66.3)		500 (63.5)
Myanmar		554 (33.6)		277 (32.5)		275 (34.9)
Others		23 (1.4)		10 (1.2)		13 (1.6)
Education	1,648		850		790	
Above high school		498 (30.2)		285 (33.5)		210 (26.6)
High school		259 (15.7)		125 (14.7)		133 (16.8)
Primary school		210 (12.7)		99 (11.6)		109 (13.8)
Secondary school		161 (9.8)		72 (8.5)		88 (11.1)
Never attended school		520 (31.6)		269 (31.7)		250 (31.7)
Occupation	1,651		852		791	
General laborer/Freelance		496 (30.0)		231 (27.1)		261 (33.0)
Customer service representative		406 (24.6)		264 (31.0)		139 (17.6)
Employees of companies/private organization		235 (14.2)		114 (13.4)		121 (15.3)
Cleaning staff/Housekeeper		77 (4.7)		60 (7.0)		17 (2.1)
Public transport driver		50 (3.0)		12 (1.4)		38 (4.8)
Trading/merchant		38 (2.3)		21 (2.5)		17 (2.1)
Others^a^		349 (21.2)		150 (17.6)		198 (25.0)
Currently work status^b^						
In a place with colleagues	1,561	1,470 (94.2)	812	765 (94.2)	742	698 (94.1)
In contact with customers	1,562	912 (58.4)	813	523 (64.3)	741	383 (51.7)
Remotely	1,550	79 (5.1)	805	37 (4.6)	737	40 (5.4)
Outdoors	1,553	914 (58.9)	806	445 (55.2)	739	466 (63.1)
Self-employed	1,534	453 (29.5)	797	241 (30.2)	729	210 (28.8)
Household income has significantly decreased due to the COVID-19 crisis	1,643	1180 (71.8)	850	651 (76.6)	785	524 (66.8)

Note:

^a^including students, good/food delivery driver, farmers, municipal worker, civil servants, security guard, medical personnel, unemployed, self-employed/personal business, state enterprise employee, tour guide, university staff, and other.

^b^Participant can answer more than 1 category; N, Number of participants; IQR, Interquartile range; BMI, Body Mass Index.

**Table 2:** Baseline characteristics related to health and risk-exposure of study population.

Baseline characteristics	Total		Female		Male	
	N	median (IQR) or n (%)	N	median (IQR) or n (%)	N	median (IQR) or n (%)
**1. Health information**						
Weight (kg)	1,644	60 (52–68)	847	55 (50–63)	789	63 (56–73)
Body Mass Index (kg/m2)	1,637	22.9 (20.5–25.8)	844	22.9 (20.5–26.0)	785	22.9 (20.6–25.6)
BMI classification	1,637		844		785	
Under weight (<18.5)		128 (7.8)		66 (7.8)		61 (7.8)
Normal weight (18.5–24.9)		1,018 (62.2)		517 (61.3)		496 (63.2)
Overweight (25.0–29.9)		360 (22.0)		192 (22.7)		166 (21.1)
Obesity (≥30)		131 (8.0)		69 (8.2)		62 (7.9)
**2. Recent travel**						
Traveled by plane or by bus within the last 2 weeks	1,649	49 (3.0)	850	31 (3.7)	791	18 (2.3)
**3. History of medical conditions**						
History of medical conditions	1,642	197 (6.8)	847	88 (10.4)	787	107 (13.6)
History of medical conditions classification*						
High blood pressure/ cardiovascular disease/ treatment for a heart-related condition	1,646	146 (8.9)	849	67 (7.9)	789	78 (9.9)
Diabetes	1,649	48 (2.9)	851	20 (2.4)	790	28 (3.5)
Ever had cancer	1,651	8 (0.5)	852	3 (0.4)	791	5 (0.6)
Respiratory disease	1,650	8 (0.5)	852	5 (0.6)	790	3 (0.4)
Chronic kidney disease on dialysis	1,651	4 (0.2)	852	1 (0.1)	791	3 (0.4)
Chronic liver disease	1,650	19 (1.2)	852	6 (0.7)	790	13 (1.7)
HIV Infection	1,651	10 (0.6)	852	2 (0.2)	791	7 (0.9)
Immunosuppressive therapy	1,649	14 (0.9)	851	6 (0.7)	790	8 (1.0)
**4. Symptoms**						
Symptoms within the last few days	1,646	358 (21.8)	848	179 (21.1)	790	177 (22.4)
Symptoms classification*						
Runny	1,650	172 (10.4)	851	76 (8.9)	791	94 (11.9)
Sore throat	1,651	108 (6.5)	852	63 (7.4)	791	45 (5.7)
Fatigue	1,649	94 (5.7)	851	49 (5.7)	790	45 (5.7)
Mouth or throat currently dry	1,650	82 (5.0)	851	47 (5.5)	791	35 (4.4)
Out of breath	1,649	44 (2.7)	850	27 (3.2)	791	17 (2.2)
Coughed	1,651	72 (4.4)	852	38 (4.5)	791	34 (4.3)
Experienced a loss of taste or smell	1,650	21 (1.3)	852	13 (1.5)	790	8 (1.0)
Diarrhea	1,651	25 (1.5)	852	15 (1.8)	791	10 (1.3)
Confused	1,650	40 (2.4)	851	19 (2.2)	791	21 (2.7)
Sneezed	1,651	81 (4.9)	852	42 (4.9)	791	39 (4.9)
Fever	1,651	38 (2.3)	852	18 (2.1)	791	20 (2.5)
Difficulties to eat or drink	1,651	5 (0.3)	852	2 (0.2)	791	3 (0.4)

Note:

*Participant can answer more than 1 category, therefore the sum of the percentages may exceed 100%; N: Number of participants; IQR: Interquartile range; BMI: Body Mass Index.

### Prevalence of SARS-CoV-2 RNA and anti-SARS-CoV-2 positivity

None of the 1,651 participants were found positive for SARS-CoV-2 RNA in nasopharyngeal/throat swab samples (0%, 95% CI: 0–0.2%) (Table 3). Fourteen (0.9%, 95% CI: 0.5–1.4%) participants were positive for anti-SARS-CoV-2 antibodies, including 7 positives for IgM and 7 positives for IgG (0.4%, 95% CI: 0.2–0.9) (Table 3).

**Table 3:** Prevalences of SARS-CoV-2 RNA and anti-SARS-CoV-2 positivity.

COVID-19 test results (N = 1,651)	n	% Positive (95% CI)
SARS-CoV-2 RNA positive	0	0 (0–0.2)
SARS-CoV-2 IgM or IgG antibodies positive	14	0.9 (0.5–1.4)
• SARS-CoV-2 IgM antibody positive	7	0.4 (0.2–0.9)
• SARS-CoV-2 IgG antibody positive	7	0.4 (0.2–0.9)

### Factors associated with SARS-CoV-2 antibody positivity

Univariable and multivariable analysis of anti-SARS-CoV-2 IgM or IgG antibodies positivity are described in Table 4. The only variable associated with SARS-CoV-2 antibody positivity was the age. The rate of anti-SARS-CoV-2 antibody positivity was higher in the older groups, as compared to the youngest group (p = 0.046). Multivariable analysis revealed that having age >50 years old remained independently associated with anti-SAR-CoV-2 antibodies positivity (OR: 5.8, 95% CI: 1.0–32.1%, p = 0.045) after adjusting with variable “Having symptoms reated-COVID-19 within the last few days” and “Occupations” (Table 4).

**Table 4:** Factors associated with isolated anti-SARS-CoV-2 antibodies.

Characteristics	Category	Anti-SARS-CoV-2 positive /Number of tested (%)	Univariable		Multivariable	
			OR (95% CI)	*p*-value	aOR (95% CI)	*p*-value
Age	≤30	2/609 (0.33)	1.00			
	31–40	5/491 (1.02)	3.12 (0.60–16.16)	***0*.*175***	2.74 (0.53–14.25)	0.232
	41–50	3/333 (0.90)	2.76 (0.46–16.60)	0.268	2.41 (0.40–14.60)	0.338
	>50	4/218 (1.83)	5.67 (1.03–31.19)	***0*.*046***	5.78 (1.04–32.10)	***0*.*045***
Sex	Female	8/852 (0.94)	1.00			
	Male	5/791 (0.63)	0.67 (0.22–2.06)	0.486		
Residence	Chiang Mai	10/1,161 (0.86)	1.00			
	Lamphun	2/417 (0.48)	0.55 (0.12–2.54)	0.448		
	Other provinces	1/23 (4.35)	5.23 (0.64–42.66)	0.122		
Country of birth	Thailand	10/1,070 (0.93)	1.30 (0.40–4.15)	0.661		
	Myanmar	4/554 (0.72)	1.00			
	Other countries	0/23 (0.00)	N/A			
Education	Above high school/ High school	7/757 (0.92)	1.00			
	Primary school/ Secondary school	4/371 (1.08)	1.17 (0.34–4.01)	0.806		
	Never attended school	3/520 (0.58)	0.62 (0.16–2.42)	0.492		
Occupations*	Less contact with people	5/290 (1.72)	1.00			
	Moderate contact with people	6/826 (0.73)	0.42 (0.13–1.38)	***0*.*151***	0.43 (0.13–1.44)	0.173
	Most contact with people	3/535 (0.56)	0.32 (0.08–1.35)	***0*.*122***	0.31 (0.07–1.34)	0.117
Work in a place with colleagues	No	1/91 (1.10)	1.00			
	Yes	13/1,470 (0.88)	0.80 (0.10–6.21)	0.833		
Work in contact with customers	No	6/650 (0.92)	1.00			
	Yes	8/912 (0.88)	0.95 (0.33–2.75)	0.924		
Traveled within the last 2 weeks	No	13/1,600 (0.81)	1.00			
	Yes	1/49 (2.04)	2.54 (0.33–19.83)	0.373		
Symptoms within the last few days	No	13/1,288 (1.01)	1.00			
	Yes	1/358 (0.28)	0.27 (0.04–2.11)	***0*.*214***	0.30 (0.04–2.29)	0.243

Note: OR: Odds ratio; aOR: Adjusted odds ratio; CI: Confidence interval.

*Occupation with less contact with people includes Cleaning staff/Housekeeper, Farmers, Unemployed, and other; Occupation with moderate contact with people includes Employees of companies/private organization, Students, Municipal worker, Civil servants, Self-employed/Personal business, General laborer/Freelance, Lecturer / Staff University, State enterprise employee, and Security guard; Occupation with most contact with people includes Customer service representative, Public transport driver, Trading/merchant, Good/food delivery driver, and Tour guide.

## Discussion

During November 2020–January 2021, we found no active SARS-CoV-2 infection among at-risk populations living in Chiang Mai and Lamphun provinces, and a seroprevalence of anti-SARS-CoV-2 IgM/IgG antibodies of 0.9%. Age over 50 years old was the only factor independently associated with exposure to the virus. Information of prevalence and seroprevalence are scarce among these at-risk asymptomatic/mild symptomatic individuals in Thailand. To our knowledge, this is a first report of prevalence and seroprevalence in at-risk populations in Northern region of Thailand during the early phase of COVID-19 endemic from November 2020–January 2021. The extremely low prevalence of SARS-CoV-2 infection in this population reflects the small outbreak (cumulative number of infected cases was about 3,400, August 31, 2020) in the country before this study commenced [14]. The low seroprevalence of anti-SARS-CoV-2 IgM/IgG antibodies indicates that few individuals were exposed to virus and thus a large number of individuals in these provinces were susceptible to SARS-CoV-2 infection. However, a short time frame between the symptom onset and antibody testing may lead to undetectable antibody results [25, 26]. However, a study conducted among hospitalized patients in Siriraj hospital, Bangkok province reported a SARS-CoV-2 prevalence of 7.5% during the February–April 2020 outbreak [20]. In addition, anti-SARS-CoV-2 IgM seroprevalence among asymptomatic staff working in community hospitals throughout the country and having close contact with infected patients during the outbreak was found 0.8–5.5% and IgG seroprevalence was 0.0–5.0% [21–23]. These IgM or IgG seroprevalence tend to be slightly higher than our results which mat reflect the higher exposure risk of health care workers in contact of COVID-19 patients. Analysis of factors associated with SARS-CoV-2 seropositivity revealed that being over 50 years old was independently and significantly associated with exposure to the virus. Our finding was consistent with the higher seropositivity rate reported in a group of individuals older than 65 years old in China [27]. Several hypotheses may explain this age effect: 1) elderly people may be more susceptible than younger people to any infection due to comorbid conditions or less active immune system, 2) they may be exposed to coronaviruses due to a higher number of hospital visits or number of family contacts, and 3) elderly people may be less concerned by the COVID-19 and don’t use personal protection as necessary [28].

Our study shows that, during the period of study, the virus was not circulating much in Chiang Mai and Lamphun provinces, Thailand. Indeed, the prevalence of positive antibodies among people with potential risk exposure to SARS-CoV-2 was very low. We believe that at that time people were scared and followed the strict preventive measure guidelines including wearing a mask, washing hands often and maintaining social distance to avoid SARS-CoV-2 infection. This compliance has contributed to successfully control COVID-19 spread during that period [29]. Nevertheless, since June 2021 the number of SARS-CoV-2 infected people has steadily increased in Thailand with over 18,000 cases/day, as data on July 31, 2021 [30]. Similarly, the situation of covid-19 pandemic worldwide has not improved. According to the recent statistics of the World Health Organization (WHO), 31 July 2021, the cumulative number of cases reported globally is nearly 194 million. It is necessary to continue an active surveillance of new COVID-19 cases and verify exposure to the virus to better control the outbreak and identify the population in urgent need for vaccination. Other preventive measures such as following strictly the COVID-19 preventive measures “DMHTT”—Distancing, Mask wearing, Hand washing, Testing, Thai Chana contact tracing apps- exercises and consumption of healthy food which can be benefits to enhance host immunity [31], would be regularly applied.

Our study had some limitations. First, we used a variety of rapid test kits to determine anti-SARS-CoV-2 IgM/IgG antibodies due to the difficulty to access the test kits during the first outbreak. Of note, the determination of anti-SARS-CoV-2 seroprevalence using immunochromatography assay may result in some false positive and false negative results. However, these kits had been approved by the Thai Food and Drug Administration (Thai FDA) to be used in routine since they are easy to use and can provide rapid results. Due to their low cost, they are suitable for large-scale studies. The sensitivity of testing also depends on time onset of disease, amount of blood-circulating antibodies. Second, we may have missed a few cases since SARS-CoV-2 RNA was not detectable in some infected individuals during their window period of infection or when the number of virus is very low in individuals at pre-symptomatic/asymptomatic phase resulting a false-negative. False-negative SARS-CoV-2 RNA detection may also occur due to other factors including pre-analytical factors (specimen collection and transportation) and inaccurate diagnostic test. It may account for a lower sensitivity. However, these problems can be avoided/prevented by strictly following the SARS-CoV-2 identification guidelines, reviewing the FDA tests evaluations, and using a combination of epidemiologic evidence and testing [32, 33]. Third, this is a cross-sectional observational study. A long-term study is needed to determine the value of both SARS-CoV-2 markers in the estimation of infection rate in the future. Finally, the number of people aged over 50 years old was relatively small as compared to other age groups which may have overestimated the seropositivity rate.

## Conclusion

This study provides data of prevalence and seroprevalence of SARS-CoV-2 infection in Chiang Mai and Lamphun provinces, Thailand, from November 2020–January 2021. The prevalence and seroprevalence were very low in these two provinces and showed a very high proportion of people were susceptible to SARS-CoV-2 infection. Individuals above 50 years old may have been more exposed to the virus. Our results indicate that vaccination against SARS-CoV-2 is urgently needed and implemented into the population, particularly in elderly people. Since SARS-CoV-2 infection remains currently a major global health concern, continuing an active surveillance for a new COVID-19 case outbreak and monitoring the immune responses are needed to better control outbreaks.

## Supporting information

S1 Table (XLS)
